# An Efficient Synthesis Strategy for Metal-Organic Frameworks: Dry-Gel Synthesis of MOF-74 Framework with High Yield and Improved Performance

**DOI:** 10.1038/srep28050

**Published:** 2016-06-16

**Authors:** Atanu Kumar Das, Rama Sesha Vemuri, Igor Kutnyakov, B. Peter McGrail, Radha Kishan Motkuri

**Affiliations:** 1 Center for Molecular Electrocatalysis, Physical Sciences Division, Pacific Northwest National Laboratory (PNNL), Richland, Washington 99352, USA.; 2 Energy and Environment Directorate, Pacific Northwest National Laboratory (PNNL), Richland, WA 99352, USA

## Abstract

Vapor-assisted dry-gel synthesis of the metal-organic framework-74 (MOF-74) structure, specifically Ni-MOF-74 produced from synthetic precursors using an organic-water hybrid solvent system, showed a very high yield (>90% with respect to 2,5-dihydroxyterepthalic acid) and enhanced performance. The Ni-MOF-74 obtained showed improved sorption characteristics towards CO_2_ and the refrigerant fluorocarbon dichlorodifluoromethane. Unlike conventional synthesis, which takes 72 hours using the tetrahydrofuran-water system, this kinetic study showed that Ni-MOF-74 forms within 12 hours under dry-gel conditions with similar performance characteristics, and exhibits its best performance characteristics even after 24 hours of heating. In the dry-gel conversion method, the physical separation of the solvent and precursor mixture allows for recycling of the solvent. We demonstrated efficient solvent recycling (up to three times) that resulted in significant cost benefits. The scaled-up manufacturing cost of Ni-MOF-74 synthesized via our dry-gel method is 45% of conventional synthesis cost. Thus, for bulk production of the MOFs, the proposed vapor-assisted, dry-gel method is efficient, simple, and inexpensive when compared to the conventional synthesis method.

Metal-organic frameworks (MOFs) have attracted much attention during the last two decades because of their enormous structural and chemical diversity in terms of high surface area, pore volumes, high thermal and chemical stabilities, and variety of pore dimensions/topologies^1^,^2^,^3^,^4^,^5^. These properties have made them superior to other traditional porous materials, and interest in their use for applications in gas/vapor sorption, molecular separation, and heterogeneous catalysis has increased significantly^1^,^6^,^7^,^8^,^9^,^10^,^11^,^12^,^13^. In spite of their tremendous potential, near-term prospects for commercial applications remain quite limited because of the lack of technologies and processes for synthesizing these materials in quantities required for industrial applications and at a low cost. Facile synthesis of MOFs is very important for lowering the cost and also for achieving fundamental understanding and viable applications. The general synthesis methodology for MOFs is very similar to molecular sieve synthesis, and usually involves hydrothermal or solvo-thermal crystallization of dissolved reactants in suitable solvents using conventional heating methods and autogeneous pressures^14^,^15^. Recent reports of alternative methods include the use of microwave^16^, sonication^17^, mechano-chemical^18^,^19^,^20^, and electrochemical synthesis^21^ methods, but all of these methods have issues with scalability, consistency, and cost that prevent their use for practical applications.

## Background

Recently, syntheses of porous materials from dry-gels have attracted considerable attention. The synthesis of porous material using the dry-gel conversion (DGC) method has potential advantages such as minimum waste disposal and reduced reactor size. In the literature, there are many reports about using steam-assisted DGC methods for synthesis of zeolites^22^,^23^,^24^, but very few reports about synthesis of MOFs^25^,^26^,^27^. Shi *et al*. synthesized zeolite imidazole framework (ZIF) materials such as ZIF-8 and ZIF-67 by replacing dimethylformamide (DMF) with water as the solvent^28^. Ahmed *et al*. reported synthesis of iron-based MIL-100(Fe) without adding any hydrofluoric (HF) acid to the reaction mixture^29^. Later, Kim *et al*. reported the synthesis using water as solvent and HF acid as an additive of the same MIL (Materials Institut Lavoisier) family of MOFs, MIL-101(Cr), which exhibited increased surface area^30^. All of the reported MOFs and ZIFs synthesized thus far using the DGC method involve synthesis chemistry that has a reaction precursor, especially in the case of organic ligand, that is water soluble at reaction temperatures. In the case of Ni-MOF-74, the organic ligand 2,5-dihydroxyterepthalic acid (DHTA) is insoluble in water, which mandates the use of an organic solvent in the solvent mixture. Therefore, the important technical challenge is to demonstrate dry-gel synthesis using hybrid organic-water solvents that have variable boiling points and vapor pressures. To the best of our knowledge, synthesis of the MOF-74 family with the DGC method using hybrid organic-water solvent mixtures has not been reported so far. Here we report the synthesis of Ni-MOF-74 structures in an organic solvent-water mixture using a vapor-assisted DGC synthesis method for the first time. Among all of the MOF materials reported to date, the microporous MOF-74 (CPO-27 or M-DOBDC) structure shows promise for gas sorption applications because of its high density of open metal centers. MOF-74 is typical of MOFs that have a high density of accessible, open metal sites, which have shown remarkable host-guest interactions leading to high storage capacities for CO_2_, CH_4_, H_2_S, xenon, fluorocarbons, etc.^31^,^32^,^33^. In this paper, we report on work that focused on synthesizing Ni-MOF-74 using the DGC method. Our results show higher yields, faster kinetics of formation, and improved performance over materials produced using typical batch synthesis methods.

### Synthesis methodology and experimental details

**Figure 1:**
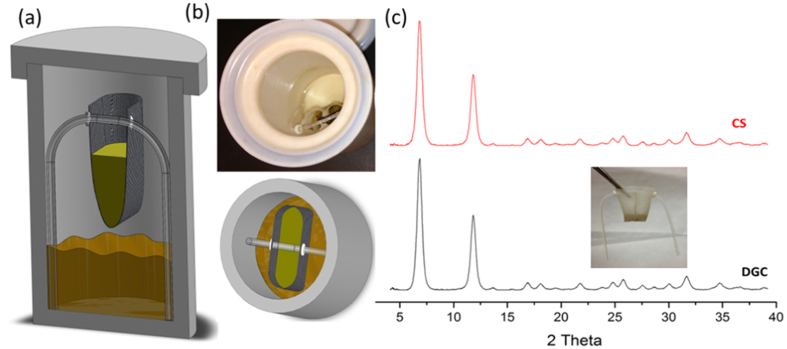
(**a**) The schematics of the vapor-assisted DGC method showing the solvent, FEP pouch, and MOF reagents – side view; (**b**) phtographs of autoclave top view and the Ni-MOF-74 containing FEP pouch; (**c**) the powder X-ray diffraction (PXRD) pattern of Ni-MOF-74 synthesized by the DGC method compared to conventional heating.

In general, M-MOF-74 (M = Ni, Co) was synthesized mainly under solvo-thermal conditions using two different approaches: 1) a tetrahydrofuran (THF)-water (1:1) mixture at 110 °C for 3 days and 2) a DMF-ethanol-water (1:1:1) mixture at 100 °C for 24–66 hours^34^,^35^,^36^. Initially, we attempted to synthesize Ni-MOF-74 using a THF-based procedure whereby the reagents 2,5-dihydroxyterepthalic acid (DHTA) and metal acetate (metal = nickel or cobalt) in a 1:2 molar ratio were ground together and then placed in a pouch made from fluorinated ethylene propylene (FEP) polymer mesh. We chose FEP polymer over the robust polytetrafluoroethylene (PTFE) material because its melt-processability using conventional heating facilitates making the pouches, and it exhibits robust characteristics similar to PTFE. The MOF precursor mixture loaded in the FEP pouch was carefully placed in a Teflon liner containing the solvent mixture (THF-water, 3 mL each), all of which was placed carefully at the bottom of the reactor as shown in Fig. 1.

The reactor was sealed and allowed to heat at 110 °C for 3 days^35^. After the heating period, the dry solid material was washed with fresh THF solvent to remove any unreacted starting material because an excess of metal salt was used. The product indicated successful formation of the MOF-74 honeycomb structure (now on MOF-74(DGC)) with an improved yield of 90%. Moreover, the liquid mixture at the bottom of the reactor after synthesis was clear and similar to the starting mixture (Electronic Supplementary Information [ESI], Figure S1), unlike the dark brown solvent mixture found after conventional synthesis. Successful formation of the Ni-MOF-74 honeycomb structure via the DGC method was verified by PXRD analysis, which revealed a match for the relative intensity and peak positions of the crystallographic data^37^. For comparison, Ni-MOF-74 also was synthesized in parallel using a conventional solvo-thermal (CS) method in which the precursors were dissolved in a THF/water mixture and heated held at 110 °C for 72 hours. The product of this synthesis (hereafter referred to as Ni-MOF-74(CS) produced an overall yield of ~65%. The PXRD analysis of the MOF-74(DGC) sample revealed an exact match to that of the MOF-74-CS sample of the honeycomb network (Fig. 1c).

To check the purity of the reagents and to verify that no mechano-chemical reaction occurred before performing the DGC synthesis, freshly ground reagents (DHTA and the metal salt) also were subjected to PXRD measurements, which showed only starting materials, and no MOF-74 peaks were observed (ESI, Figure S4). Thermogravimetric analysis (TGA) performed on Ni-MOF-74 synthesized by the DGC method showed a weight loss of 18 to 23% as temperature was increased from 25 to 200 °C, which corresponds to the loss of solvent molecules and is comparable to weight loss experienced during the conventional synthesis method (ESI, Figure S6). Brunauer–Emmett–Teller (BET) surface area analysis was performed for both Ni-MOF-74(DGC) and Ni-MOF-74(CS) samples at 77 K using N_2_ adsorption; the Ni-MOF-74(DGC) showed a high surface area of ~1350 m^2^/g, while the conventional heating sample showed a surface area of ~1029 m^2^/g, which is in line with the values reported in the literature ( ESI, Figures S7–S10)^37^. It is important to note that the MOF-74 synthesized using the DGC method was used “as is” with simple THF washing, while conventional Ni-MOF-74(CS) was tested after multiple solvent activation steps using methanol soaking for 3 days and replacing the methanol every 24 hours.

## Results and Discussion

### Adsorption performance

**Figure 2:**
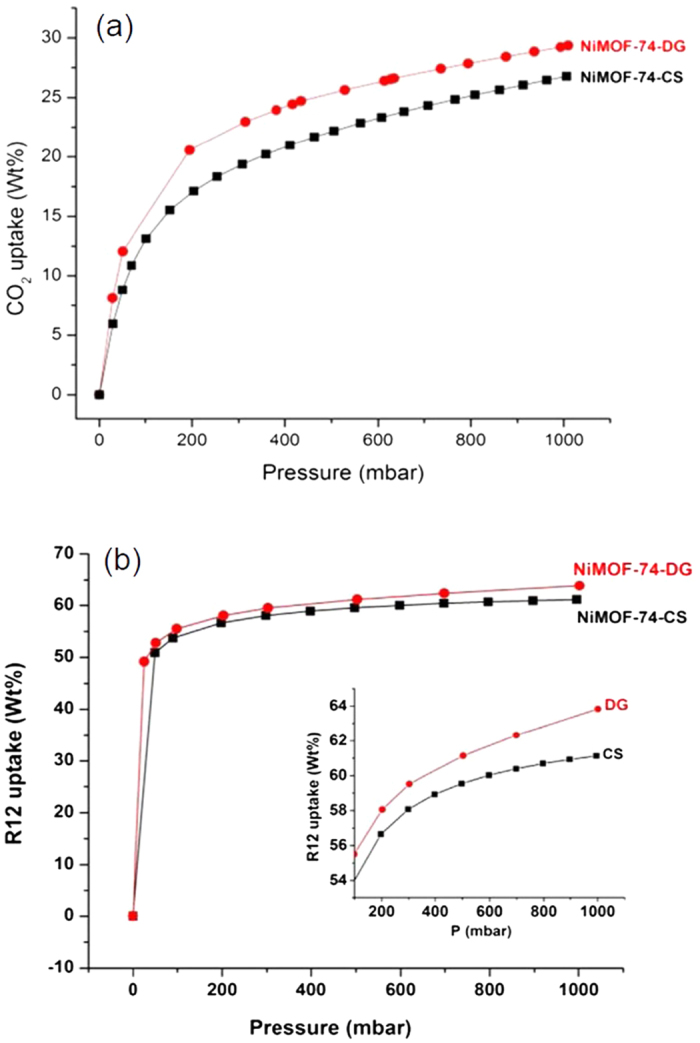
Adsorption and desorption studies of CO_2_ (**a**) and fluorocarbon dichlorodifluoromethane (R12) (**b**) in Ni-MOF-74(DGC) and Ni-MOF-74(CS). Note that there are improved sorption capacities in both CO_2_ and R12 sorption measurements. For clarity, R12 sorption capacities are presented in a zoomed-in scale to see the improved performance more clearly (inset).

Ni-MOF-74 is known to be a promising candidate for low-pressure CO_2_ sorption applications; therefore, we tested the sorption characteristics of both the DGC and CS samples. Both MOF samples were subjected to the same activation procedure before testing their sorption capabilities. Our CO_2_ isotherm for the Ni-MOF-74(CS) is very similar to published data within the experimental error. It is interesting to see that the Ni-MOF-74(DGC) showed enhanced CO_2_ adsorption performance up to 9% (2.5 wt%) as shown in Fig. 2.

Though it is a smaller number, the enhanced CO_2_ sorption capacity was observed throughout the pressure curve from 100 to 1000 mbar (Fig. 2a). To further elucidate the enhanced sorption capacities of the DGC method, we extended the adsorption towards R12 because Ni-MOF-74 showed extremely high sorption capacities at low pressures (50.8 wt% at 100 mbar)^38^. We attempted the same R12 adsorption studies for both DGC and CS samples at room temperature. Similar to the CO_2_ sorption studies, the DGC method showed enhanced sorption capacities, which is close to an ~4.5% increase in R12 adsorption characteristics over Ni-MOF-74(CS) as shown in Fig. 2b. Enhanced sorption capacities are observed throughout the adsorption curve, and for clarity, enhanced sorption can be clearly seen from the enlarged portion of the curve (Fig. 2, inset).

Further, to understand the optimized synthesis conditions and to further appreciate the capability of the DGC method over the solvo-thermal synthesis method, we performed a time-dependent kinetics study of Ni-MOF-74 synthesis using the DGC method. In this study, Ni-MOF-74(DGC) synthesis was carried out at variable time durations of 72, 48, 24, and 12 hours under identical thermal and reaction conditions. The PXRD results from all the samples showed the formation of the honeycomb network structure. More surprisingly, the Ni-MOF-74(DGC) synthesized after just 12 hours of heating time also showed successful formation of the honeycomb network (Figure S3, ESI). The BET surface area measurements on these samples reveals that increasing the heating time improves the overall surface area of the material, but not much improvement is observed between the 48- to 72-hour heating time (Table 1). This result was further confirmed by testing the CO_2_ sorption performance on the samples where Ni-MOF-74(DGC)-48 h and Ni-MOF-74(DGC)-72h showed similar sorption characteristics (ESI, Figures S11–15). This result implies that the optimal time duration of the DGC synthesis was less than the conventional synthesis method. Similarly, we successfully extended the DGC method to the synthesis of cobalt MOF-74 using the THF-water solvent system. The synthesis and XRD results of Co-MOF-74(DGC) samples were identical to samples produced by solvo-thermal synthesis. The results are shown in Figure S5 (ESI).

To further demonstrate the vapor-assisted DGC method, we also attempted a DMF-based synthesis procedure developed by the Matzger group for synthesizing the Ni-MOF-74 structure^34^. The MOF precursors (DHTA and nickel nitrate in a 1:3.33 molar ratio) were prepared by grinding them together and then loading the ground mixture in an FEP pouch that was carefully placed in a Teflon liner containing a solvent mixture (DMF-ethanol-water, 2 mL each). Similarly, the reactor was sealed and allowed to heat at 100 °C for 24 hours according to the procedure. The resulting Ni-MOF-74 also had the MOF-74 honeycomb structure (ESI, Figure S2). Interestingly, although the DMF boiling point (~156 °C) was considerably higher than the reaction temperature, ethanol and water vapors can carry DMF to the MOF reagents where it acts as a catalyst for removing the proton from the organic acid for forming the MOF-74 structure^39^.

MOF-74 structures can be successfully formed in the vapor phase of the DGC method in an organic-water hybrid solvent. The high yield with improved performance might result from a solid-vapor reaction in which no contact exists between the solid reactants and liquid solvent during MOF formation. Thus, unwanted side reactions that generally occur in the liquid phase can be avoided, resulting high purity of MOF and enhanced yield.

**Figure 3:**
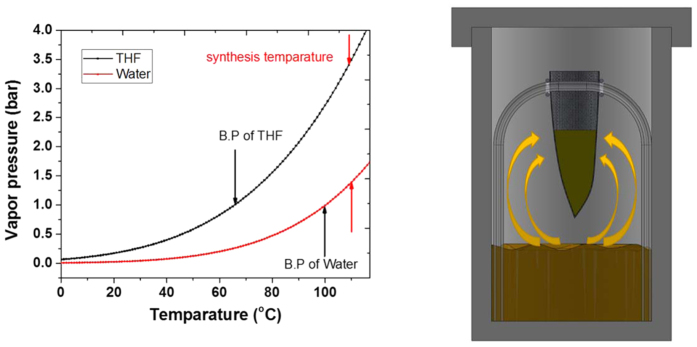
Left: Pressure vs. temperature (vapor pressure curve) plot of water and THF compared with the reaction conditions. Right: Cross-sectional view of the dry-gel apparatus inside the autoclave. Solvent vapors penetrate through the porous FEP pouch from all directions.

Regarding the crystallization process in steam-assisted ZIF-8 and also in zeolites, the solvent water heated to 110 °C under autogeneous pressure in the autoclave can generate a pressure close to 1.8 bar^28^. Fig. 3 shows the vapor pressure curves for both water and THF.

We used Antoine’s equation to derive the vapor pressure curve.


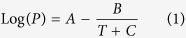


In this equation, A, B, C are solvent dependent parameters, and values are obtained from NIST database^40^,^41^. The synthesis temparature is well above the boiling points of both water and THF. From the vapor pressure curve, the vapor pressures of THF and water, at a synthesis temparature of 110 °C, are 3.49 bar and 1.39 bar, respectively. At this temperature and these pressures, the phase of the water is at the liquid-vapor boundary while the THF is in the vapor phase. Hence, both of components of the THF-water solvent mixture are transported in the vapor phase to the precursor mixture (nickel salt+DHTA ligand) and penetrate it. Our novel porous FEP polymer pouch bag allows uniform penetration of the solvent vapor mix over the entire salt-ligand mixture compared to the previously reported use of a ceramic cup in which the opening of the cup limits solution transport. The solvent vapor, which is transported to the salt-ligand mixture, condenses and is adsorbed on the precursor particles, creating a “solution-like” phase in which the same chemistry can occur, as in bulk solution, thereby catalyzing the MOF formation reaction^28^,^42^.

### Solvent recycling

**Figure 4:**
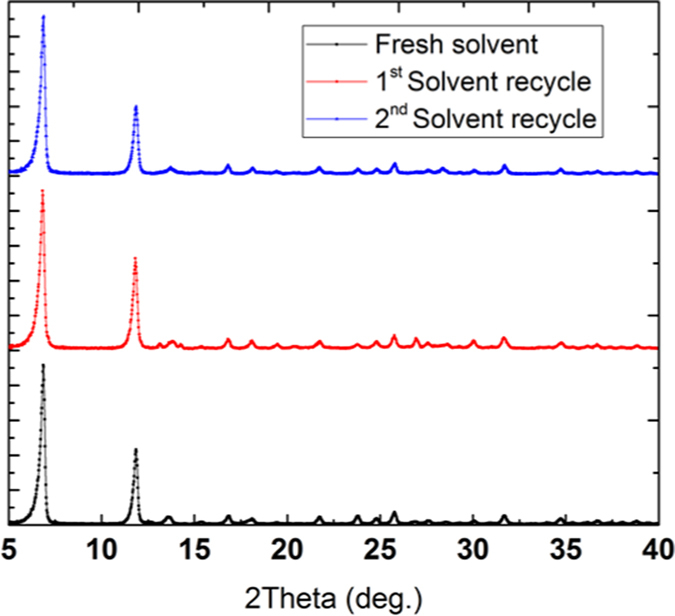
Powder XRD patterns of the Ni-MOF-74(DGC) synthesized using recycling the solvent over three cycles.

In the dry-gel method the solvent-precursor mixture is physically separated. Moreover, the solvent is transported to precursor mixture in the vapor phase, leaving behind the particulate impurities. Therefore, we thought that the solvent could be recycled without compromising the purity of the synthesized MOF. To verify the solvent reusability, we recycled the same solvent over three synthesis cycles. For cycle 1, we started with a pure solvent mixture (1:1 THF:water ratio, 10 mL) in an autoclave. We loaded the MOF-74 precursor mixture in the FEP pouch and carefully placed it in a Teflon liner containing the solvent mixture (THF-water, 5 mL each); the autoclave was heated at 110 °C for 24 hours. When the reaction was complete, we carefully removed the FEP pouch from the autoclave without disturbing the solvent. For cycle 2, another FEP pouch containing freshly prepared precursor mixture was placed in the autoclave with the recycled solvent. The synthesis process was continued at 110 °C for 24 hours. The same procedure is repeated for third cycle. Figure 4 shows the PXRD results of the Ni-MOF-74(DGC) synthesized in three cycles using recycled solvent where the XRD patterns indicate the formation of MOF-74 honeycomb network. The absence of impurity peaks and x-ray background is indicative of the high purity of the Ni-MOF-74 produced using recycled solvent.

Because the solvent is an important cost contributor in bulk MOF synthesis, we performed a cost analysis for scaling up Ni-MOF-74 synthesis using the DGC method with recycled solvent (see ESI Section III). We found the general manufacturing cost of synthesizing Ni-MOF-74 using the conventional method to be $6,523/kg. When synthesis using the new DGC method was analyzed, the manufacturing cost was less than half (i.e., ~$2881/kg) with the solvent recycled for at least three times. The price might decrease even further by increasing the number of cycles (Tables S1 and S2). Thus, the DGC method showed a clear cost advantage over the conventional synthesis method and the method can be easily extended to other methods.

In conclusion, we successfully synthesized high-performance Ni-MOF-74 using a hybrid organic-water solvent mixture via a DGC method. In comparison to the Ni-MOF-74 produced using the CS method, the dry-gel Ni-MOF-74 showed a higher surface area and improved gas-capture performance for both CO_2_ and R12. We also demonstrated that Ni-MOF-74 can be synthesized in 24 hours via the DGC method, and the resulting product exhibits acceptable purity and performance characteristics. Also, physical separation of the solvent mixture from the precursor mixture minimizes unwanted side reactions, thus allowing clean solvent mixture remaining after synthesis to be recycled multiple times. This implies an environmental and cost benefit in MOF synthesis. Overall, we demonstrated a technical advance in MOF synthesis in terms of the synthesis time scale, improved performance and raw material recycling that has important implications for low-cost manufacturing of MOF structures.

## Additional Information

**How to cite this article**: Das, A. K. *et al.* An Efficient Synthesis Strategy for Metal-Organic Frameworks: Dry-Gel Synthesis of MOF-74 Framework with High Yield and Improved Performance. *Sci. Rep.* **6**, 28050; doi: 10.1038/srep28050 (2016).

## Supplementary Material

Supplementary Information

## Figures and Tables

**Table 1:** Ni-MOF-74 obtained with DGC and CS synthesis conditions and their surface areas and yields.

Material	Method and Solvents	Temperature (°C)	Time (h)	BET surface area (m^2^/g)	Yield
Ni-MOF-74(DGC)	DGC (TW)	110	72	1350	90.1%
Ni-MOF-74(CS)	CS (TW)	110	72	1029	65.2%
Ni-MOF-74(DGC)	DGC (TW)	110	48	1291	89.3%
Ni-MOF-74(DGC)	DGC (TW)	110	24	1049	87.0%
Ni-MOF-74	DGC (TW)	110	12	1041	82.1%
Ni-MOF-74(DGC)	DGC (DEW)	100	24	1063	72.1%
Ni-MOF-74(CS)	CS (DEW)	100	24	983	50.2%

DGC: Dry-gel conversion; CS: conventional synthesis; TW: THF + water (1:1).

DEW: DMF + ethanol + water (1:1:1).
