# Supplementary material for: An Efficient Synthesis Strategy for Metal-Organic Frameworks: Dry-Gel Synthesis of MOF-74 Framework with High Yield and Improved Performance
Source: Sci Rep. 2016 Jun 16;6:28050. doi: 10.1038/srep28050 (PMC4910056; doi:10.1038/srep28050)
Supplement: Supplementary Information [file srep28050-s1.pdf]

Supporting information

An Efficient Synthesis Strategy for Metal-organic Frameworks: Dry-Gel Synthesis of MOF-74 Framework with High Yield and Improved Performance

Atanu Kumar Das,[‡] Rama Sesha Vemuri, [†] Igor Kutnyakov,[‡] B. Peter McGrail,[†] Radha Kishan Motkuri,[*,†]

[†] *Energy and Environment Directorate, Pacific Northwest National Laboratory (PNNL), Richland, WA 99352, USA.*
[‡] *Fundamental and Computational Sciences Directorate, PNNL, Richland, WA 99352, USA.*

*To whom correspondence: Radhakishan.Motkuri@pnnl.gov

Table of Contents:

Section I: Material synthesis and Characterization

1. **Dry-gel synthesis of Ni-MOF-74 (Ni-MOF-74(DGC))**
2. **Conventional solvothermal synthesis of Ni-MOF-74 (Ni-MOF-74(CS))**
3. **Dry-gel synthesis of Co-MOF-74 (Co-MOF-74(DGC))**

Section II: Gas adsorption studies

Section III: Cost analysis of solvent recycling using DGC method

Section I: Material synthesis and characterization

Dry-gel synthesis of Ni-MOF-74 (Ni-MOF-74(DGC))

0.374 g (1.5 mmol) of nickel(II) acetate tetrahydrate and 0.148 g (0.75 mmol) of H_2DHTA (2,5-dihydroxyterepthalic acid were mixed (metal: ligand = 1:2) and ground well to get fine mixture. The mixture was placed in a holed polymer mesh pouch made from Fluorinated Ethylene Propylene polymer (FEP) and it was placed in a Teflon-lined stainless steel autoclave. 10 ml of solvent mixture of THF: H_2O (1:1) or DMF: EtOH: H_2O, was put at the bottom of the reactor. The entire assembly was heated at 110 °C for different time span (72h, 48h, 24h and 12h) for THF-water system while 24h heating for DMF-ethanol-water system respectively. After the reaction, compound was collected and washed thoroughly with THF (2 –3 times) to remove unreacted starting material. The solvent was collected as colorless liquid.

Conventional solvothermal synthesis of Ni-MOF-74 (Ni-MOF-74(CS))

0.374 g (1.5 mmol) of nickel(II) acetate tetrahydrate and 0.148 g (0.75 mmol) of H_2DHTA (2,5-dihydroxyterepthalic acid were dissolved in 10 mL of a mixed solvent (THF:H2O = 1:1) and the mixture was placed in a Teflon-lined stainless steel autoclave. The entire assembly was heated at 110 °C for 72h. After the reaction, compound was collected and soaked in methanol for 3 days with replacing fresh methanol every 24h.

Dry-gel synthesis of Co-MOF-74 (Co-MOF-74(DGC))

0.476 g (1.88 mmol) of cobalt(II) acetate tetrahydrate and 0.186 g (0.94 mmol) g of H_2DHTA (2,5-dihydroxyterepthalic acid were mixed (metal: ligand = 1:2) and ground well to get fine mixture. The mixture was placed in a holed polymer mesh pouch made from Fluorinated Ethylene Propylene polymer (FEP) and it was placed in a Teflon-lined stainless steel autoclave. 10 ml of solvent mixture of THF: H_2O (1:1) was put at the bottom of the reactor. The entire

assembly was heated at 110 °C for 72h. After the reaction, compound was collected and washed thoroughly with THF (2 –3 times) to remove unreacted starting material. The solvent was collected as colorless liquid.

Section II: Sorption studies.

Gas adsorption studies (CO_2 and R12)

For BET surface area measurements, the nitrogen adsorption and desorption isotherms were measured in a Quantachrome instrument at 77K using liquid nitrogen. Before the adsorption experiment, the MOF samples were activated at 200 °C for 12h under vacuum. The CO_2 sorption experiments were also performed using the Quantachrome instrument. The fluorocarbon (R12) adsorption, desorption measurements were performed using Intelligence Gravimetric Analyzer (IGA) instrument. Prior to measuring the sorption studies, the sample was placed in a container of the IGA chamber and the weight of the sample was recorded before activation. The temperature of the furnace was increased up to 200°C under vacuum at a rate of 5°C/min to remove the trapped solvent molecules. The sample was cooled to RT, its dry mass was set, and the experimental temperature 25°C was maintained by the IGA water bath. The static mode of the IGA was used to measure the sorption studies. The pressure points were set beforehand using the IGA software. The pressure was maintained at the set point by active computer control of the inlet/outlet valves throughout the duration of the experiment. Weight increases resulting from adsorption at each pressure step were plotted against the pressure.

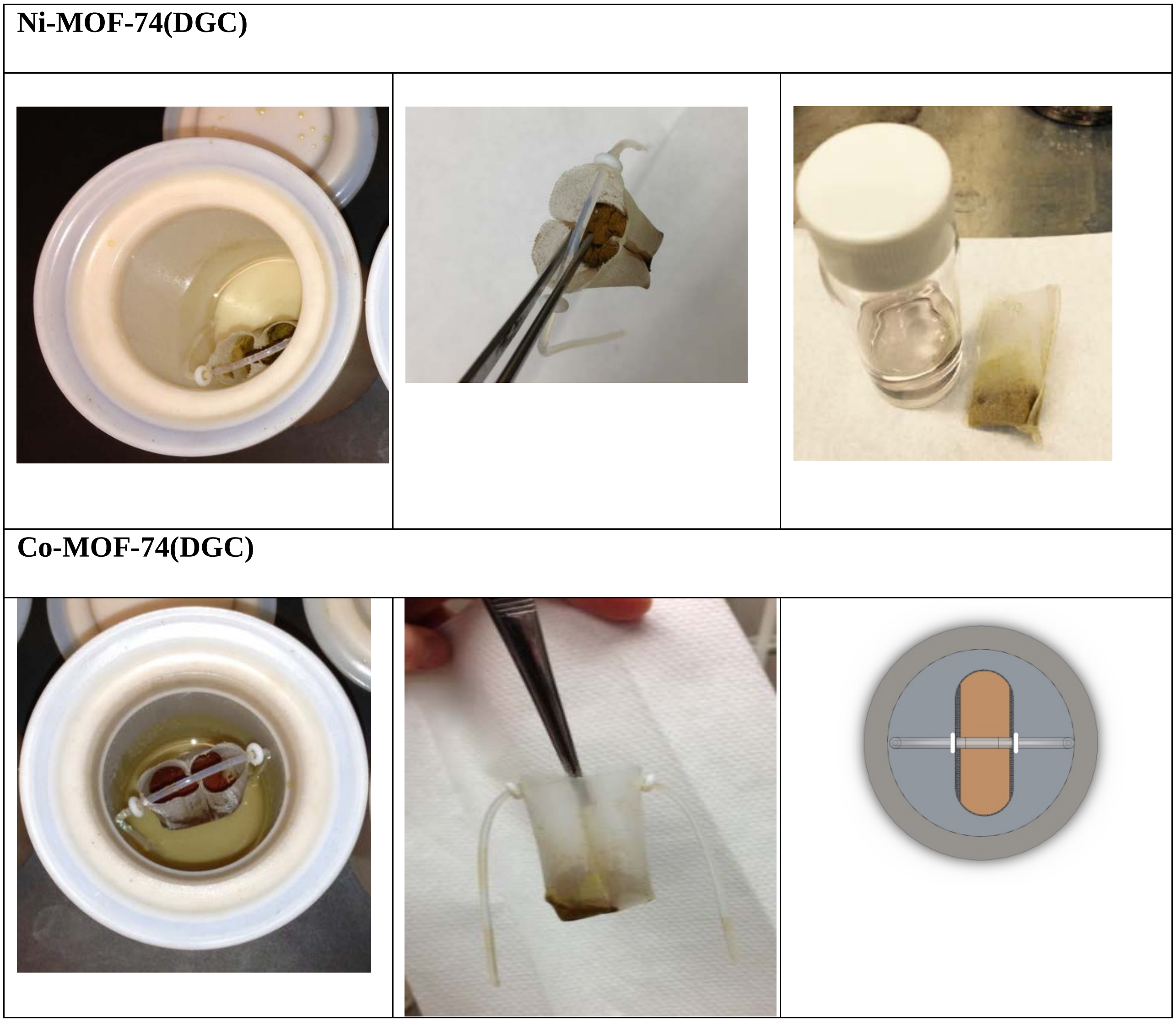

Figure S1: The Photographs of dry-gel conversion reactor after synthesis of Ni-MOF-74 and Co-MOF-74.

Section I: Characterization

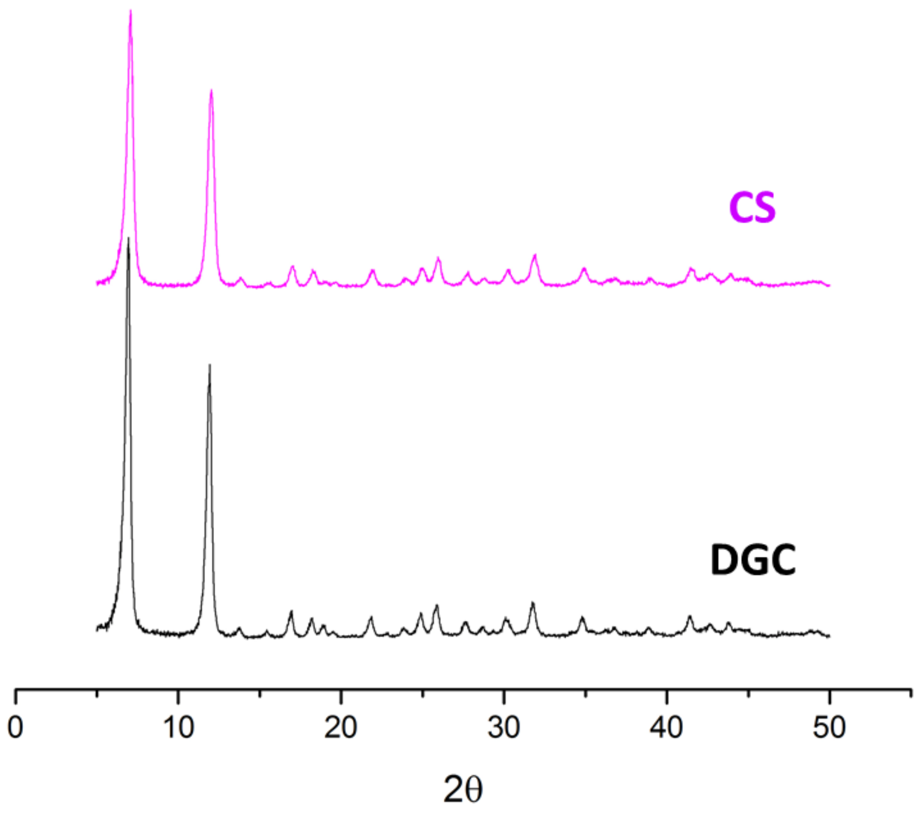

Figure S2: Powder XRD spectra of Ni-MOF-74 synthesized via two different methods (DGC and CS) using DMF-ethanol-water system at 100 °C for 24h.

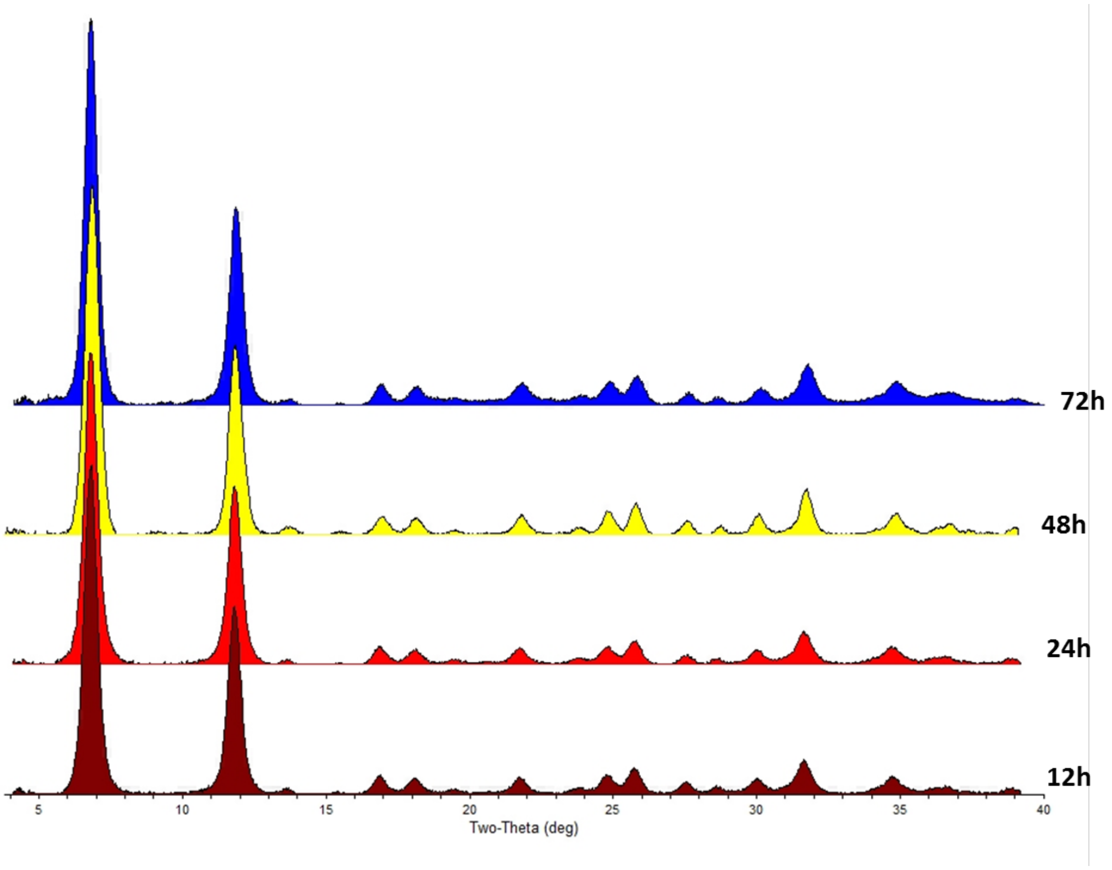

Figure S3: Powder XRD spectra of Ni-MOF-74 synthesized via DGC method at different time periods from 12h, 24h, 48h and 72h using THF-water based system 110 °C.

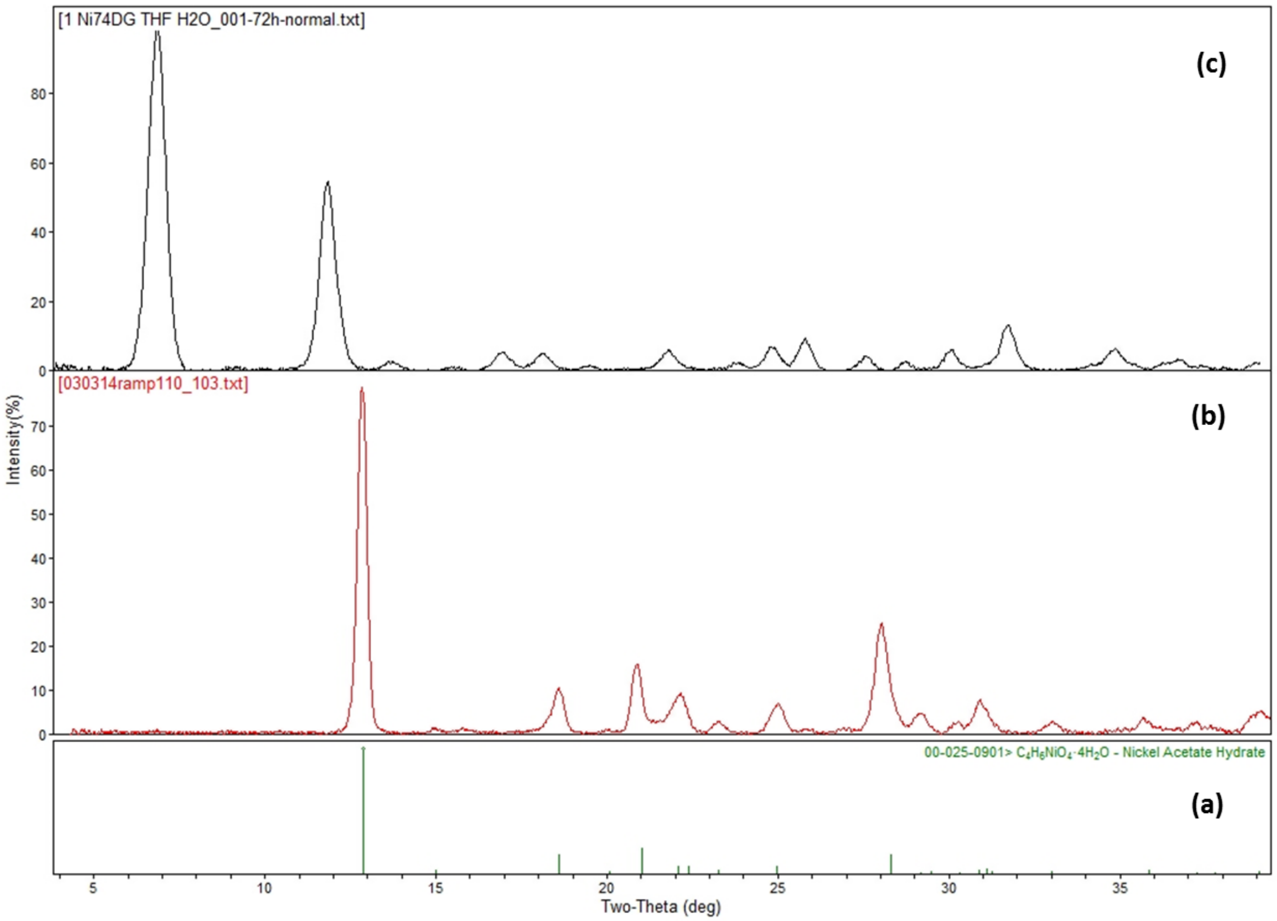

Figure S4: Comparison of powder XRD spectra of (a) Nickel acetate standard with (b) grounded DHTA + Nickel acetate hydrate before DGC and (c) Ni-MOF-74(DGC) after synthesis. Note that the grounded material (b) is only showing the nickel salt.

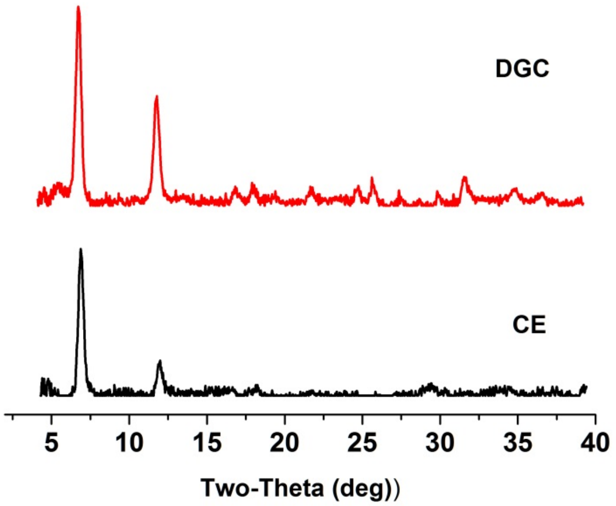

Figure S5: Powder XRD spectra of Co-MOF-74 synthesized via two different methods (DGC and CS) using THF-water based system 110 °C 72h

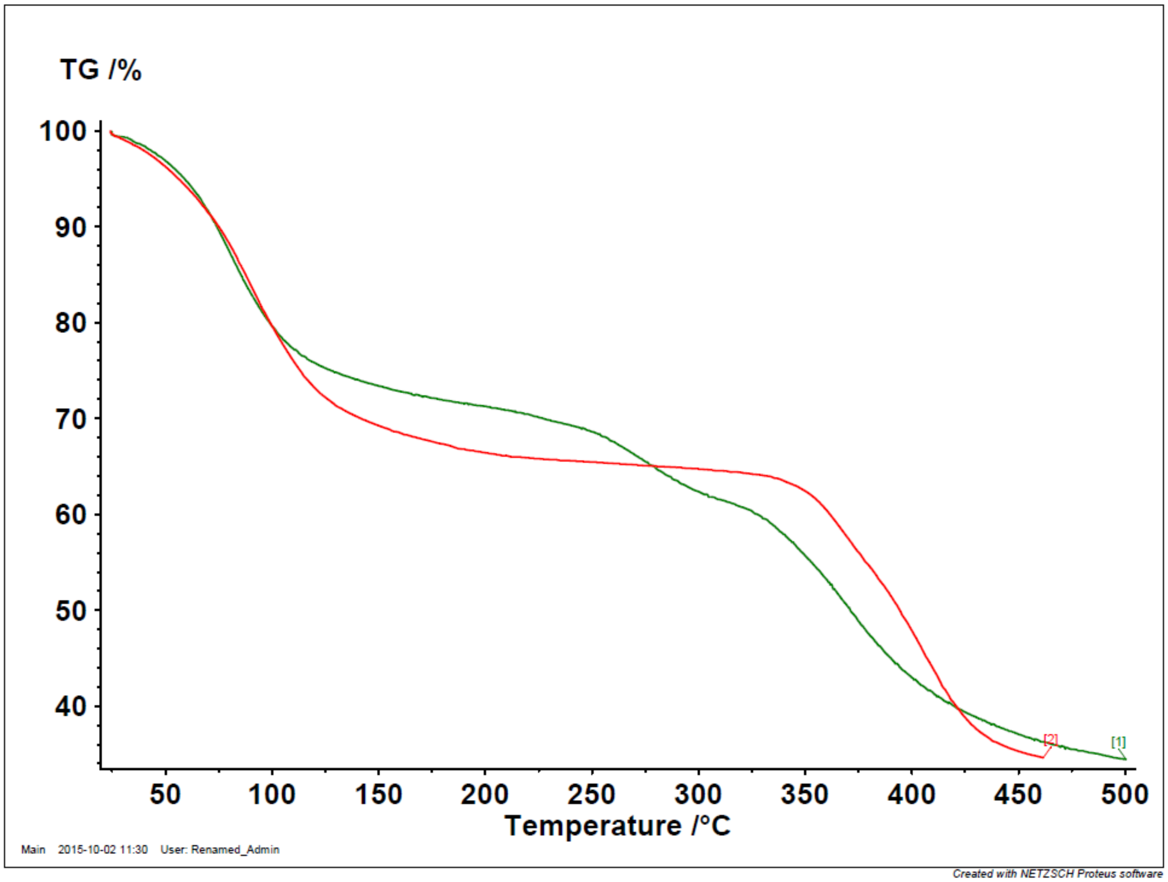

Figure S6: Comparison of the TGA curves for Ni-MOF-74 synthesized via two different methods (DGC-green and CS-red) using THF-water based system 110 °C for 72h. In CS method, the MOF sample exchanged with methanol for 72h while it washed with THF in DGC.

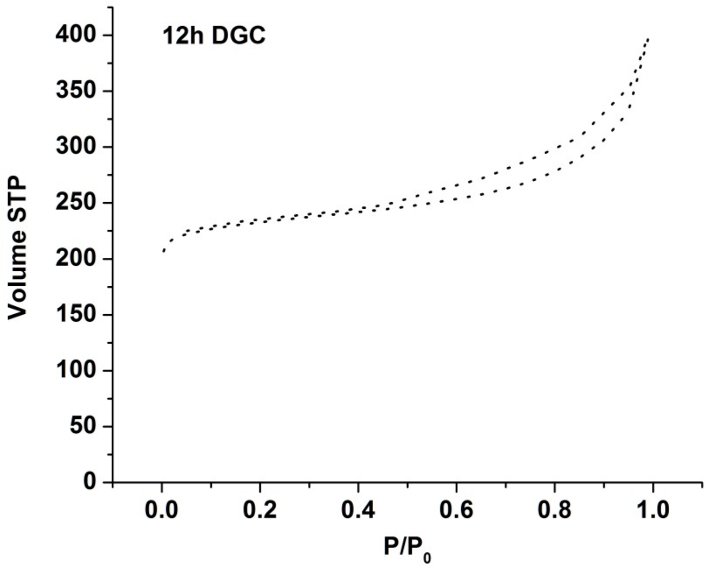

Figure S7. BET isotherm of Ni-MOF-74(DGC)-12h (THF-Water at 110 °C for 48h) at 1bar and 77K using N_2.

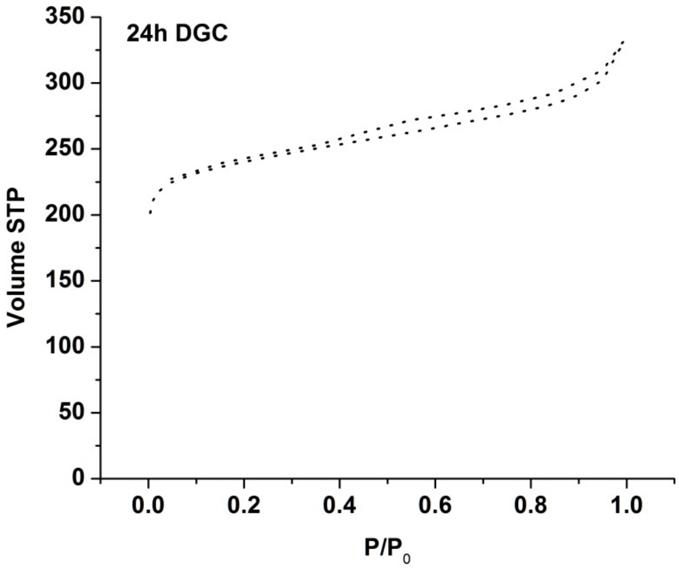

Figure S8. BET isotherm of Ni-MOF-74(DGC)-24h (THF-Water at 110 °C for 24h) at 1bar and 77K using N_2.

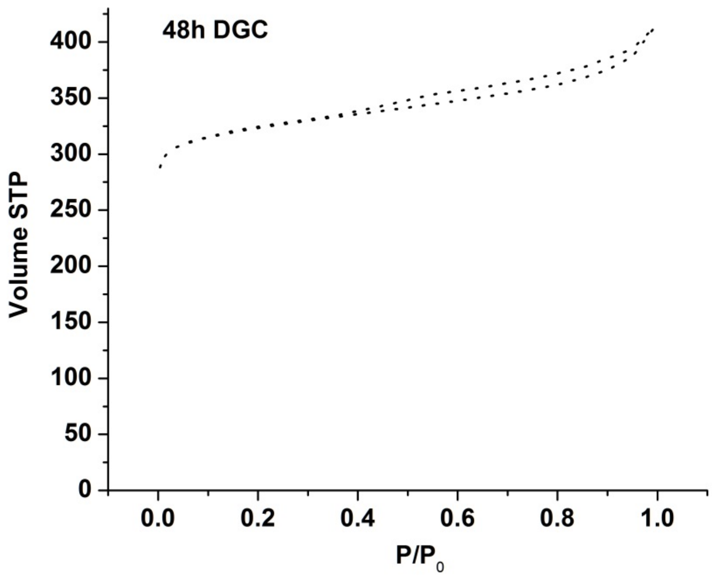

Figure S9. BET isotherm of Ni-MOF-74(DGC)-48h (THF-Water at 110 °C for 48h) at 1bar and 77K using N_2.

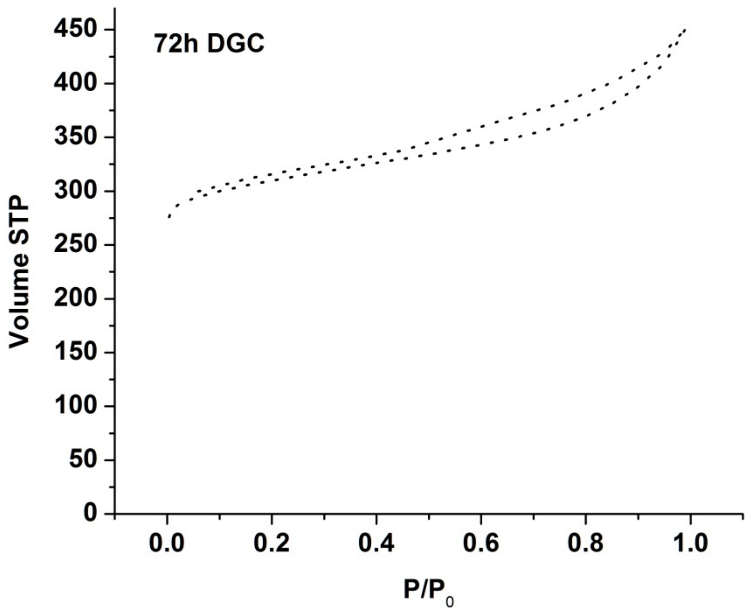

Figure S10. BET isotherm of Ni-MOF-74(DGC)-72h (THF-Water at 110 °C for 72h) at 1bar and 77K using N_2.

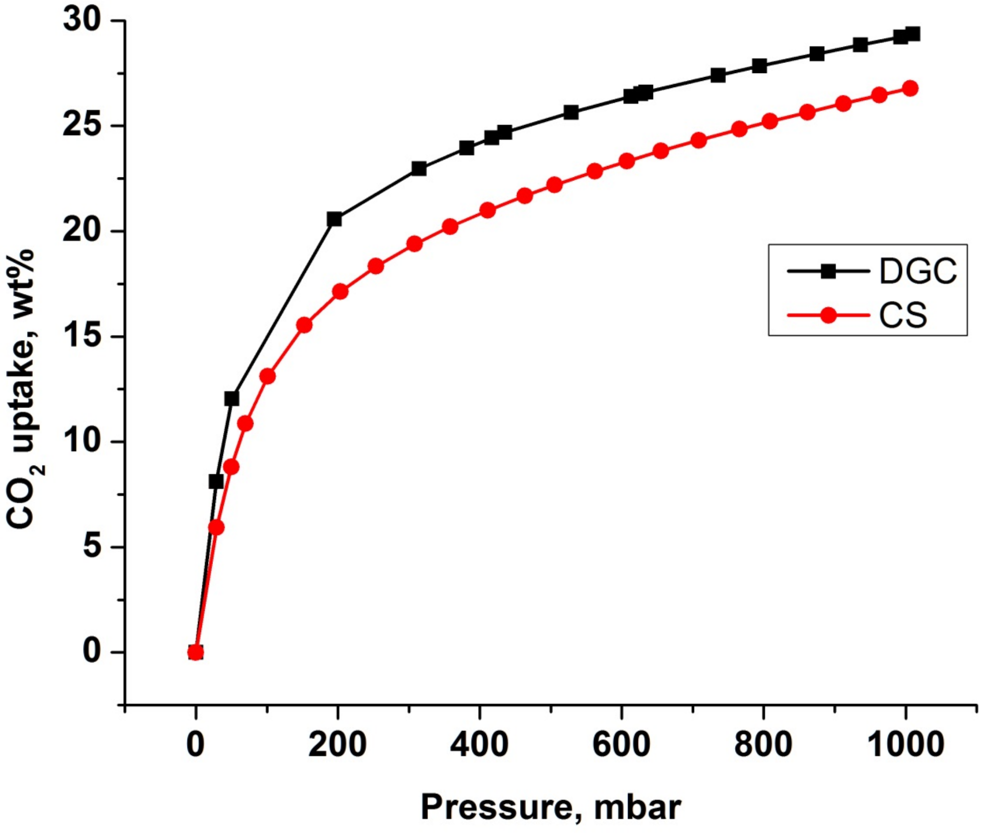

Figure S11: CO_2 sorption in Ni-MOF-74 synthesized under DGC and CS (THF-Water at 110 °C for 72h) conditions

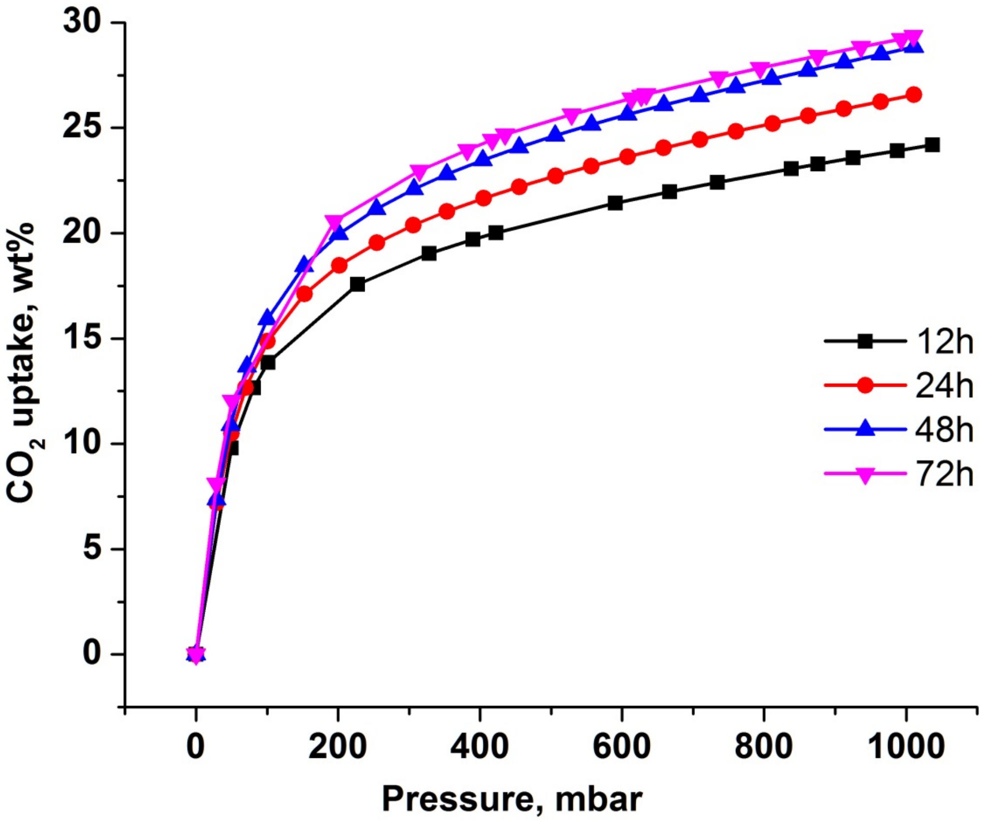

Figure S12: CO_2 sorption in Ni-MOF-74 synthesized under DGC method (THF-Water at 110 °C) with variable heating periods

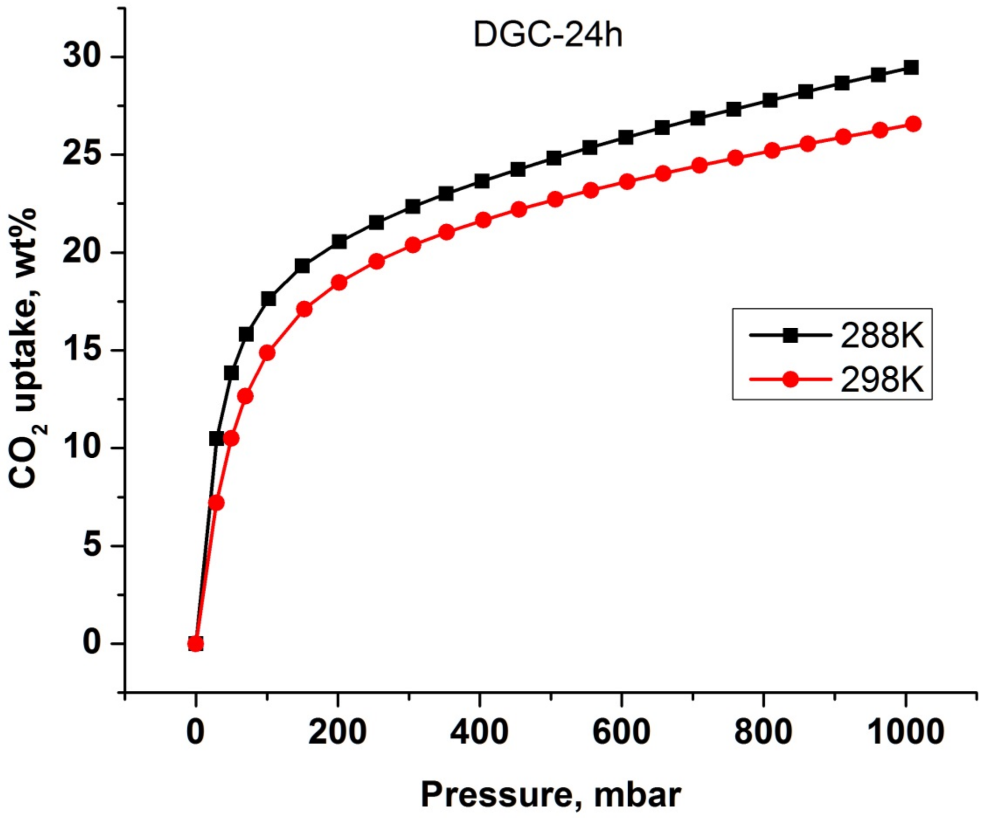

Figure S13: CO_2 Sorption and desorption at two different temperatures of 298K, 288K in Ni-MOF-74(DGC)-24h (Synthesized in THF-water system at 110 °C for 24h)

.

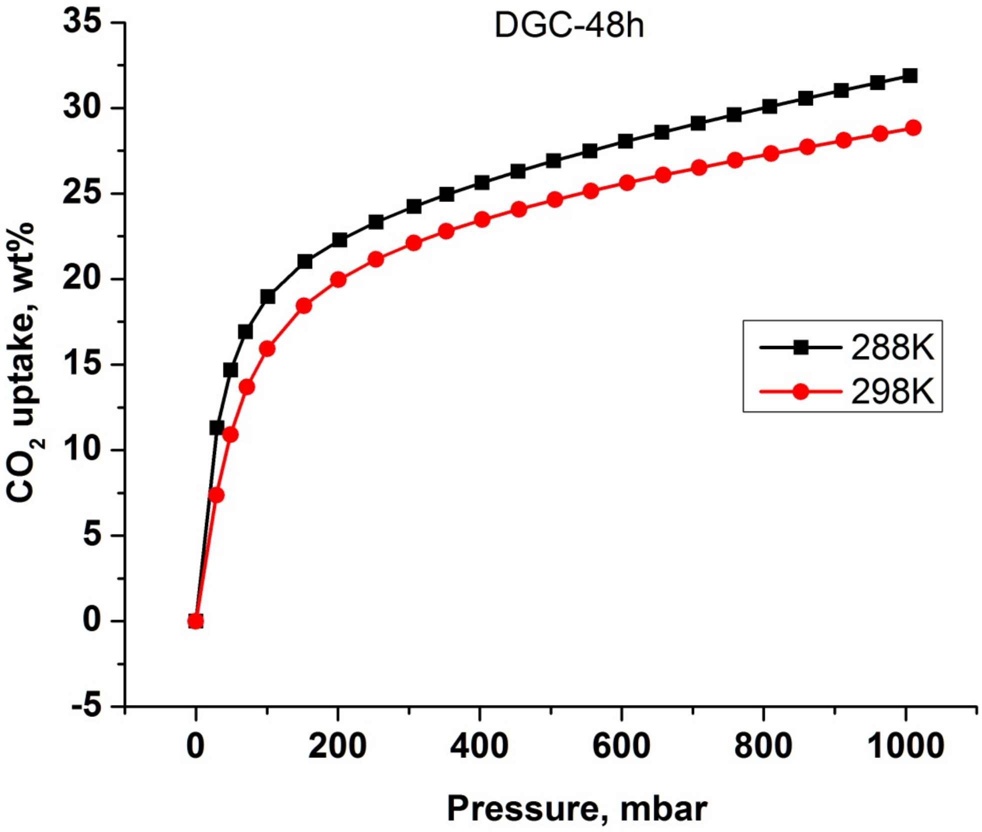

Figure S14: CO_2 Sorption and desorption at two different temperatures of 298K, 288K in Ni-MOF-74(DGC)-48h (Synthesized in THF-water system at 110 °C for 48h)

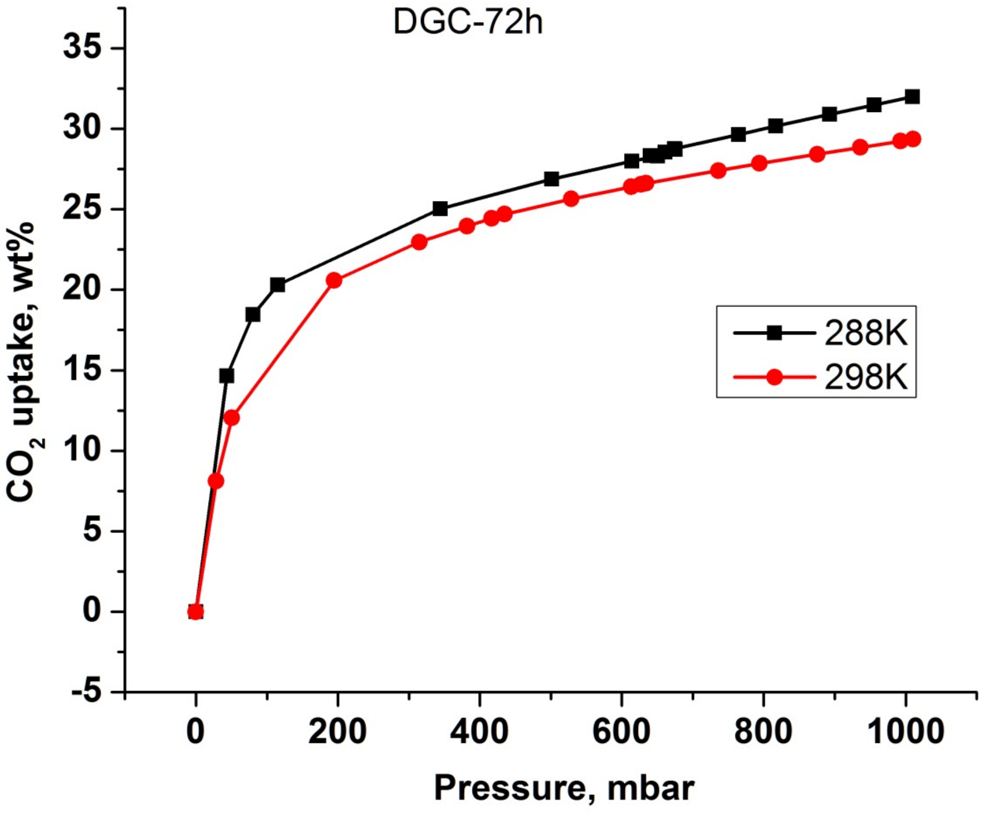

Figure S15: CO_2 Sorption and desorption at two different temperatures of 298K, 288K in Ni-MOF-74(DGC)-72h (Synthesized in THF-water system at 110 °C for 72h)

Section III : Cost analysis of solvent recycling using DGC method

Here we introduce a simplified cost analysis for the Ni-MOF-74 synthesis which elucidates the cost advantage of DGC method. The solvent mixture can be reused for subsequent DGC runs, by switching the pouch bag with fresh precursor mixture. We believe that integrity of the solvent mixture will be retained up to 3 runs, hence we cost compare the DGC method with conventional solvothermal synthesis with 3 cycles of the synthesis. **Table S1** illustrates the scaled up cost from the cost of the materials used in this run using DGC method while **Table S2** using conventional synthesis. Prices of the materials used in this context are bulk priced from the commercial vendors. The quantities of the reactants and solvents are based on the Dietzel procedure.[1]

Table S1. Cost of Ni-MOF-74 synthesis using dry-gel method.

	Chemical	Reaction weight	Scaled up Weight	Cost/kg or Cost/L	Total price of material	Total
Cycle#1	2,5 dihydroxyterepthalic acid (DHTA)	0.148g	1Kg	$1400	$1400	$4240
	Nickel acetate	0.373g	2Kg	$120	$240	
	THF	5ml	26 L	$100	$2600	
Cycle#2	DHTA	0.148g	1Kg	$1400	$1400	$1640
	Nickel acetate	0.373g	2Kg	$120	$240	
	THF	5ml	26L	$0 (solvent recycled)	$0	
Cycle#3	DHTA	0.148g	1Kg	$1400	$1400	$1640
	Nickel acetate	0.373g	2Kg	$120	$240	
	THF	5ml	26L	$0 (solvent recycled)	$0	

Yield of material based on DHTA weight for DGC synthesis= 87% [See table.1 main text]

Ni-MOF-74 produced= 3Kg *87/100 = 2.61Kg

Cost of the Ni-MOF-74(DGC) = (4240+1640+1640)/2.61= $2881/Kg

Table S2. Cost of Ni-MOF-74 synthesis using conventional solvothermal method:

In case of solvothermal synthesis method the solvent has lot of impurities and is either discarded or purified for reuse (which may be costly)

	Chemical	Reaction weight	Scaled up Weight	Cost/kg or Cost/L	Total price of material/Kg	Total
Cycle#1	2,5 dihydroxyterepthalic acid (DHTA)	0.148g	1Kg	$1400	$1400	$4240
	Nickel acetate	0.373g	2Kg	$120	$240	
	THF	5ml	26 L	$100	$2600	
Cycle#2	DHTA	0.148g	1Kg	$1400	$1400	$4240
	Nickel acetate	0.373g	2Kg	$120	$240	
	THF	5ml	26L	$2600	$2600	
Cycle#3	DHTA	0.148g	1Kg	$1400	$1400	$4240
	Nickel acetate	0.373g	2Kg	$120	$2420	
	THF	5ml	26L	$2600	$2600	

Yield of material based on DHTA weight for DGC synthesis= 65% [See table.1 main text]

Ni-MOF-74 produced= 3kg *65/100 = 1.95Kg

Cost of the Ni-MOF-74(CS) = (4240+4240+4240)/ 1.95= $6523/Kg

1. (a) Dietzel, P. D. C.; Johnsen, R. E.; Fjellvag, H.; Bordiga, S.; Groppo, E.; Chavan, S.; Blom, R., Adsorption properties and structure of CO(2) adsorbed on open coordination sites of metal-organic framework Ni(2)(dhtp) from gas adsorption, IR spectroscopy and X-ray diffraction. *Chem Commun* **2008,** (41), 5125-5127; (b) Dietzel, P. D. C.; Panella, B.; Hirscher, M.; Blom, R.; Fjellvag, H., Hydrogen adsorption in a nickel based coordination polymer with open metal sites in the cylindrical cavities of the desolvated framework. *Chem Commun* **2006,** (9), 959-961.
